# Supplementary material for: Validity concerns with the Revised Study Process Questionnaire (R-SPQ-2F) in undergraduate anatomy & physiology students
Source: PLoS One. 2021 Apr 29;16(4):e0250600. doi: 10.1371/journal.pone.0250600 (PMC8084138; doi:10.1371/journal.pone.0250600)
Supplement: Attachment — Submitted filename: Response Letter.pdf [file pone.0250600.s002.pdf]

SOUTHERN WESLEYAN UNIVERSITY | Division of Science

Staci Johnson, MS, PhD
907 Wesleyan Drive, PO Box 1020
Central, SC 29630
864-644-5256
✉ *sjohnson@swu.edu*

Dear Journal Editor and Reviewers,

December 21, 2020

Please find attached the documents required for the resubmission of our article titled "Validity concerns with the Revised Study Process Questionnaire (R-SPQ-2F) in Undergraduate Anatomy & Physiology Students" (PONE-D-20-22094). Our research team has carefully reviewed the comments and suggestions. We appreciate the time and insights provided by these comments and believe the manuscript is now stronger with incorporation of many of these suggestions. The table below provides details about how we have responded to each point from the reviewer.

**Reviewer Comments/ Suggestions**	**Author Response**
I suggest the authors to shorten the following sections (and evaluate to integrate discussion): Development of SAL theory (shorten lines 16-44; evaluate to delete lines 45-53)	Section has been shortened to mainly discuss the work of Biggs, while acknowledging the foundational work of Marton, Entwistle, and Pask.
I suggest the authors to shorten the following sections (and evaluate to integrate discussion): Development of Surface and Deep Approaches to Learning (lines 76-88)	This section has been shortened.
I suggest the authors to shorten the following sections (and evaluate to integrate discussion): Analysis (delete lines 237-244; shorten lines 254-271)	This section has been shortened to the extent possible without compromising the clarity of the analysis description.
I suggest the authors to shorten the following sections (and evaluate to integrate discussion): Examples of Analysis (the section is very long. Evaluate to shorten in not more than 20-25 lines)	We have removed one example of analysis to shorten the section. However, we believe a complete description of all steps is important for clarity of our process.
I suggest the authors to shorten the following sections (and evaluate to integrate discussion): Model specification (lines 563-573 should be maybe moved to data analysis)	This section is already part of the larger analysis section. This section has followed best-practice reporting according to Jackson[1].

(continued)

Reviewer Comments/ Suggestions	Author Response
I suggest the author to spend some lines in a small review of specific recent studies regarding medical students. I feel that the authors should add 20 new recent literature contributes regarding medical students at least and new theoretical perspectives (e.g. Variation theory and others) regarding student approaches.	This study has specifically studied undergraduate students enrolled in an anatomy & physiology course. We recognize that additional work has been done with this instrument and medical students, but that is beyond the scope of our work. This work is intended to highlight the lack of previous information about undergraduates in this context.
In my opinion one aspect lacking is the contents concerning the professional teaching practice and the implications and applications of the instrument.	Restructuring and additions to the Discussion section have attempted to make more clear that the instrument requires additional validity and reliability testing in new contexts before it should be used in future work with the context of undergraduate anatomy & physiology. Because of this, a note about possible future work related to teaching practice has been added.
Discussion is short and not did fully discuss results.	We've added more to the Discussion to specifically highlight the results and how these findings cause us to question the reliability and validity of this instrument for the current population.
The research question s too limited. Please, try to better develop a rationale and a literature review that better explain the research gap that the study wants to fill in and the research questions.	Some additional information has been added to the "Research Question" section to more clearly explain the research question of the current study and how this fills a gap in the literature.
Try to explain why the response rate is so low.	A sentence has been added to describe the lack of incentives for completing the survey.
Since the article is very long, I try to suggest to delete some figure (figure 1, 2 and 3).	Figure 1 has been deleted. However, we feel that Figure 2 and 3 are essential for describing our mixed methods/ qualitative sample and our procedure of analysis.

We appreciate the opportunity to receive feedback and improve our manuscript. Please let me know if additional discussions are needed to move this publication forward.

Regards,

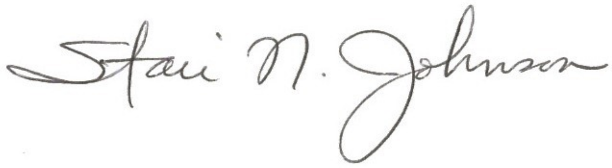

Staci Johnson, MS, PhD

References

1. Jackson DL, Gillaspy, Jr JA, Purc-Stephenson R. Reporting Practices in Confirmatory Factor Analysis: An Overview and Some Recommendations. Psychological Methods. 2009;14(1):6–23. doi:10.1037/a0014694.
