# Supplementary material for: Validity concerns with the Revised Study Process Questionnaire (R-SPQ-2F) in undergraduate anatomy & physiology students
Source: PLoS One. 2021 Apr 29;16(4):e0250600. doi: 10.1371/journal.pone.0250600 (PMC8084138; doi:10.1371/journal.pone.0250600)
Supplement: Attachment — Submitted filename: PLoS Letter 2.pdf [file pone.0250600.s003.pdf]

SOUTHERN WESLEYAN UNIVERSITY | **Division of Science**

Staci Johnson, MS, PhD
907 Wesleyan Drive, PO Box 1020
Central, SC 29630
864-644-5256
sjohnson@swu.edu

Dear Journal Editor and Reviewers,

February 8, 2021

Please find attached the documents required for our newest resubmission of our article titled "Validity concerns with the Revised Study Process Questionnaire (R-SPQ-2F) in Undergraduate Anatomy & Physiology Students" (PONE-D-20-22094R1). Our research team has carefully reviewed the comments and suggestions. We, again, appreciate the time and insights provided by these comments and believe the manuscript is now stronger with incorporation of many of these suggestions. The table below provides details about how we have responded to each point from the reviewer.

**Reviewer Comments/Suggestions**	**Author Response**
The CFA fit indices are clearly very poor and not moderate. Did you test any different model? I suggest looking at the modification indices or to exclude some items to identify a better factor solution.	Our characterization of CFA fit is based on published and accepted fit index cut-offs [1, 2, 3]. In addition, while some fit indices are worse than those reported by Justicia and colleagues, two indices (RMSEA and SRMR) were a slight improvement over those reported in their analysis and did provide some minimal evidence of reliability. We have included the citations in the manuscript (pp. 26-27) and clarified language within the Abstract (pp. 1-2) and Discussion section (pp. 27-28) to indicate that the model fits moderately at best.<br>We performed a preliminary exploratory factor analysis, and the model yielded by the 2-factor approach had acceptable factor loadings with the 20 items grouping as reported by Biggs and Justicia. This is not reported, as it is beyond the scope of this study and would increase the length of this paper. We agree that additional models should be tested and have updated the Future Work section (p. 30) to indicate this.

(continued on following page)

I'm not sure I can understand how the factors can be deemed reliable but not valid: p. 32, line 755 - "We thus have minimal evidence that the two-factor structure of the R-SPQ-2F is a moderately reasonable fit to the data for this population, which supports the R-SPQ-2F reliably measuring two distinct factors. This would agree with the findings of Stes and colleagues [By the way, Stes et al.'s findings are not described in the introduction because have been deleted]. However, in light of the results from the other components of this study, we have reason to believe that the two factors measured by the instrument may not truly represent deep and surface approaches to learning, calling into question the validity of the instrument." p. 34, line 815 - "While the reliability of the two-factor structure of the R-SPQ-2F has been deemed acceptable, questions about the validity of this instrument remain." Usually reliability is measured using Cronbach's alpha or McDonald's Omega, but this is not the case. Hence, I suggest to specify how reliability was assessed.	We thank you for catching that our reference to Stes and colleagues on longer makes sense following our edits of the Introduction. We have addressed this issue. To add clarity to the discussion of reliability and validity, we have added a section in the Introduction (p. 6) regarding validity and reliability of survey instruments. This section briefly describes important aspects of reliability (stability, internal consistency, equivalence) and validity (content validity, construct validity, criterion validity) and distinguishes them. We have then made more explicit connections between the evidence and specific aspects of reliability and validity at appropriate points in the Analysis (p. 17: Quantitative and qualitative scale comparison; Item review) and Discussion (p. 28, 29) sections. We also particularly address why we chose to carry out factor analysis rather than using Cronbach's α or Kuder-Richardson 20 to assess internal consistency of the R-SPQ-2F (p. 21). We have added appropriate citations to support these additional details.

We appreciate the opportunity to receive the additional feedback and to make improvements our manuscript. Please let me know if additional discussions are needed to move this publication forward.

Regards,

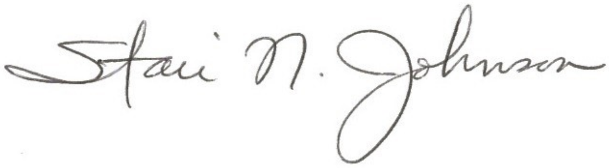

Staci Johnson, MS, PhD

References

1. Bandalos D. Measurement theory and applications for the social sciences. New York: Guilford Press; 2018.

2. Browne MW, Cudeck R. Alternative ways of assessing model fit. In: Bollen KA, Long JS, editors. Testing structural equation models. Newbury Park, CA: Sage; 1993. p. 136–162.

3. Hu L, Bentler PM. Cutoff criteria for fit indexes in covariance structure analysis: Conventional criteria versus new alternatives. Structural Equation Modeling: A Multidisciplinary Journal. 1999;6(1):1–55. doi:10.1080/10705519909540118.
