# Supplementary material for: Validity concerns with the Revised Study Process Questionnaire (R-SPQ-2F) in undergraduate anatomy & physiology students
Source: PLoS One. 2021 Apr 29;16(4):e0250600. doi: 10.1371/journal.pone.0250600 (PMC8084138; doi:10.1371/journal.pone.0250600)
Supplement: Attachment — Submitted filename: Rebuttal_Letter_3.pdf [file pone.0250600.s004.pdf]

SOUTHERN WESLEYAN UNIVERSITY | Division of Science

Staci Johnson, MS, PhD
907 Wesleyan Drive, PO Box 1020
Central, SC 29630
864-644-5256
✉ *sjohnson@swu.edu*

Dear Journal Editor and Reviewers,

April 1, 2021

Please find attached the documents required for our newest resubmission of our article titled "Validity concerns with the Revised Study Process Questionnaire (R-SPQ-2F) in Undergraduate Anatomy & Physiology Students" (PONE-D-20-22094R2). Our research team has carefully reviewed the comments and suggestions. We, again, appreciate the time and insights provided by these comments and believe our responses have strengthened the paper. The table below provides details about how we have responded to each point from the reviewer.

**Reviewer Comments/Suggestions**	**Author Response**
Whereas I can again appreciate the effort made in revising the paper, unfortunately I'm not totally convinced that the revisions help to improve the paper or to solve my previous concerns. Overall, there is still a mixed use of two distinct concepts: reliability and validity. To the best of my knowledge, reliability refers to "internal consistency" and validity refers to the "internal structure" of a scale. If the internal structure (or factor structure) is not supported by the CFA, to discuss reliability is useless.	We appreciate this description of the reviewer's concern. We are not clear about the reviewer's mention of "mixed use of ... reliability and validity". We have defined these terms in a manner consistent with education literature (pp. 6–7 of the manuscript) and described how our data provide evidence in support or not of each concept. We have added four citations ([1, 2, 3, 4]) in that section as further support for our use of these definitions. While we agree that a lack of validity for an instrument would make reliability useless for the factors purported to be measured, it is possible that the instrument is reliably measuring other constructs that have not been identified in the population of interest. This information is helpful to report in the interest of future work that might attempt to identify these factors/constructs. We have also edited the Discussion (pp. 28–30), Conclusion (p. 31), and Future Work (p. 32) sections of the manuscript to address this more explicitly.

(continued on following page)

**Reviewer Comments/Suggestions**	**Author Response**
"Our characterization of CFA fit is based on published and accepted fit index cut-offs [1, 2, 3]." The fact that similar indices have been published do not mean that they are acceptable.	The criteria used were determined prior to analysis based on the recommendations of Jackson et al. (reported on p. 28, lines 617–620) and are the cutoffs recommended in the cited textbook on measurement theory (Bandalos [5]). The supporting citations of the papers in which these cutoffs are established are referenced in the Bandalos text have been cited extensively (Hu & Bentley [6], >75,000 citations; Browne & Cudeck [7], 5800 citations). We consider this to be sufficient evidence of the acceptability of these cutoffs and ask that, if the editor disagrees, he help us understand what evidence he would like to see on this front.
ML and WLS estimation methods: fit indices are not acceptable in both cases, even if slightly better for ML. However, 11 out of 20 factor loadings are <.30 if ML estimation is used. This result confirms the inadequacy of the tested factor solution.	Based on the cutoffs selected, RMSEA and SRMR indicate acceptable model fit under the ML estimation method, and are improved values compared to those in the analysis by Justicia and colleagues, which was deemed acceptable for the model that is now used in education research. We also respectfully direct the attention of the reviewer to Figure 3, in which the factor loadings are reported. Factor loadings for all items were greater than 0.35 using the ML method.<br><br>We ultimately wish to argue that, while the fit and reliability of the model may not be *good*, they are at least as acceptable as other reliability results reported in the literature by those who have assessed this instrument in the past and with other populations. This underscores the importance of our validity concerns rather than undermining them. If *at best* the model fit is deemed acceptable and the instrument cited as reliable, the other qualitative aspects of our analysis yield sufficient concerns about validity to indicate that the survey is not measuring the constructs it is intended to measure. However, because reliability is necessary for validity, if our results do *not* provide sufficient reliability of the instrument due to the model fit indices, then our concerns about the validity of the results remain.<br><br>We have endeavored to clarify this distinction in our paper in the CFA report (pp. 21–28), Discussion (pp. 28–31), and Abstract (pp. 1–2).

(continued on following page)

Reviewer Comments/Suggestions	Author Response
At p. 6, the Section Reliability and Validity is confounding: a) "Reliability is evaluated using measures of internal consistency": why these measures were not calculated? b) reliability has nothing to do with the "equivalence to other instruments measuring the same constructs". This aspect concerns validity. c) Criterion validity and construct validity definitions are inverted (and not investigated in the current study), and it is not mentioned internal construct validity that has to do with the factorial structure of a scale, largely investigated in the paper.	a) Upon further consideration, we agree that the measures of internal consistency should be reported to strengthen the analysis section and thank the reviewer for these clarifying comments. Measures of McDonald's omega have been reported in the Confirmatory Factor Analysis section of the paper (p. 28, lines 623–626).<br><br>b) and c) We respectfully refer the reviewer to the additions we have made that are outlined above related to new citations related to terms and their associated definitions. Equivalence is regularly used in education research to establish one part of a reliability, which does overlap with validity. Since this is a point of contention between researchers about whether this should be counted as reliability or validity, we are happy to reduce confusion among potential readers by only considering under the umbrella of validity.
p. 21, line 477-481: "internal consistency or single-scale instruments. . . . For instruments intended to have multiple factors, such. . . [31]." Even in the cited paper, the point is that to determine internal consistency, we need to know the internal structure of the scale. That is, if the scale has two factors, two internal consistency values should be reported (and a global value if the factors are correlated). Specifically, McDonald's Omega is a better than Cronbach's alpha when multiple factors have been identified. I strongly suggest deleting these two added parts.	We thank the reviewer for this clarification. Coefficients omega are now reported for each factor under each CFA estimation method.
to my opinion results and discussion sections should stress that the CFA analysis did not support the intended factor structure of the scale. Thus, internal construct validity was not demonstrated. Reliability was not investigated (unless reliability indices were reported) and, however, is worthless if the scale is not valid. So I suggest to delete comments on reliability.	We have edited the abstract, discussion of reliability and validity, discussion, and conclusion to clarify that although the R-SPQ-2F is widely accepted as valid and reliable, perhaps it shouldn't be. We now emphasize that, although our reliability estimates are at least as good as those for other populations as reported in the literature, we nonetheless have considerable concern over validity. Our concerns over validity may reasonably extend to other populations as well since validity has never been sufficiently addressed in the literature for other populations either. We are particularly grateful to the editor for pushing on this point, as it strengthens the implications of the paper considerably.

We appreciate the opportunity to receive the additional feedback and to make improvements our manuscript. Please let me know if additional discussions are needed to move this publication forward.

Regards,

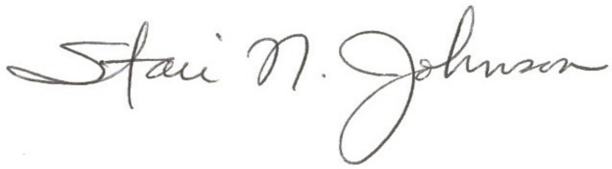

Staci Johnson, MS, PhD

References

1. Artino AR, LaRochelle JS, Dezee KJ, Gehlbach H. Developing questionnaires for educational research: AMEE Guide No. 87. Medical Teacher. 2014;36(6):463–474.

2. Rickards G, Magee C, Artino AR. You can't fix by analysis what you've spoiled by design: Developing survey instruments and collecting validity evidence. Journal of Graduate Medical Education. 2012;4(4):407–410.

3. Sullivan G. A primer on the validity of assessment instruments. Journal of Graduate Medical Education. 2011;3(2):119–120.

4. Taherdoost H. Validity and reliability of the research instrument; How to test the validation of a questionnaire/survey in a research. International Journal of Academic Research in Management. 2016;5(3):28–36.

5. Bandalos D. Measurement theory and applications for the social sciences. New York: Guilford Press; 2018.

6. Hu L, Bentler PM. Cutoff criteria for fit indexes in covariance structure analysis: Conventional criteria versus new alternatives. Structural Equation Modeling: A Multidisciplinary Journal. 1999;6(1):1–55. doi:10.1080/10705519909540118.

7. Browne MW, Cudeck R. Alternative ways of assessing model fit. In: Bollen KA, Long JS, editors. Testing structural equation models. Newbury Park, CA: Sage; 1993. p. 136–162.
